# Prevalence of Hepatitis B Virus Infection among Inmates at the Monrovia Central Prison, Liberia

**DOI:** 10.3390/tropicalmed8030139

**Published:** 2023-02-24

**Authors:** David B. Vessellee, Akua K. Yalley, David N. Adjei, Mark Appeaning, Prince N. Odoom, Ransford Kyeremeh, Abena Asefuaba Yalley, Bernice Dahn, Nicholas I. Nii-Trebi

**Affiliations:** 1 Department of Medical Laboratory Sciences, School of Biomedical and Allied Health Sciences, University of Ghana, Accra P.O. Box KB 143, Ghana; 2 College of Health Sciences, University of Liberia, Capitol Hill P.O. Box 10-9020, Monrovia 1000, Liberia; 3 Department of Medical Laboratory Science, Faculty of Health and Allied Sciences, Koforidua Technical University, Koforidua P.O. Box KF 981, Ghana; 4 Department of Biochemistry, Cell and Molecular Biology, University of Ghana, Legon, Accra P.O. Box LG25, Ghana; 5 Department of Medical Microbiology, University of Ghana Medical School, Accra P.O. Box 4236, Ghana; 6 Zukunftskolleg, University of Konstanz, 78464 Konstanz, Germany

**Keywords:** hepatitis B virus, serological profile, qPCR, viral load, prevalence, prison inmates, Liberia

## Abstract

Determination of hepatitis B virus (HBV) infections in key populations including prison inmates is crucial for formulating appropriate intervention approaches. However, in many low-income countries, such as Liberia, there is hardly any documentation on HBV prevalence among inmates. This study determined and evaluated the prevalence of HBV infections among incarcerated persons in the Monrovia Central Prison, Liberia. One hundred participants comprising 76 males and 24 females were studied. Participants’ demographic and potential risk factors information were obtained using a semi-structured questionnaire, and blood samples were collected for the analysis. Plasma was tested for five HBV serological markers, namely, HBsAg, HBsAb, HBeAg, HBeAb, and HBcAb. The seroreactivity of actively infected persons was confirmed by nucleic acid detection. Results of the serological assay showed that 34% of the participants had been exposed to the virus and 14% were actively infected. qPCR confirmed HBV DNA in seven actively infected samples. Statistical analysis indicated that a low level of education, a history of blood transfusion, and intravenous drug use, were significant predictors of active HBV infection and HBV exposure, respectively. These findings might make the testing and vaccination of convicts against HBV infection prior to their admission into prison facilities imperative.

## 1. Introduction

Hepatitis B is an infectious viral disease that mostly spreads through blood and other body fluids, and results in both chronic or acute necro-inflammatory liver diseases [1]. The hepatitis B virus currently represents an important global public health concern because of its high rate of chronic liver disease morbidity and mortality, although the majority of individuals have access to an effective vaccine [2]. Approximately 360 million people worldwide have chronic hepatitis caused by HBV, which results in 620,000 fatalities each year [3]. Of note, HBV can spread through intravenous drug use, blood and saliva, sexual contact, mother-to-child contact, and contaminated objects [4], and the levels of engagement in these risk practices with or without control measures largely influence the spread of the virus in a population.

Three zones of chronic hepatitis B virus infection prevalence have been described: namely, high (8.0%), intermediate (2–8%), and low (2% or below). In high-endemic areas, hepatitis B surface antigen (HBsAg) seropositivity is higher than 8% and about 45.0% of the world’s people live in such areas. These include Southeast Asia, the Pacific Basin, the Middle East, and a few countries in Eastern Europe, among others [5,6]. The region with the highest endemicity is Sub-Saharan Africa, where more than 8% of the population has been affected by the virus and almost fifty million of the population, especially in hyperendemic countries such as Nigeria, Namibia, Gabon, Cameroon, and Burkina Faso, are chronic HBV carriers [7]. Other countries, such as Egypt, Tunisia, Algeria, and Morocco, which are all in the north of the continent, are classified as having intermediate levels of endemicity (2–7%). In contrast, less than 2% of the population in North America, Western and Northern Europe as well as some parts of South America and Australia are hepatitis B virus carriers [8,9]. However, in Liberia, there is hardly any documentation on the prevalence of hepatitis B virus infections in the general population as well as key populations such as prisons.

Within the prison environment, regular social and physical interactions, such as intravenous drug use, among others, take place among the inmates as well as between the inmates and their officials [10]. Consequently, blood-borne diseases including HBV are generally more prevalent in prison populations than in the general population [4,11]. It is also well-known that in many prison facilities, there are common issues of overcrowding and poor infection control and prevention (IPC) systems. Liberia, for example, has 15 prisons, one in each of the country’s 15 political subdivisions, or counties. The Monrovia Central Prison (MCP) on Center Street on the southern beach side of the country’s capital, Monrovia, is the largest and most crowded prison, where the blocks were built more with functionality and security than with reform and care in focus. As of June 2022, the MCP, which was originally designed to hold 370 inmates, had a total population of 1560 (males and females) according to the prison administration. This astronomical increase in the MCP population has resulted in worsened conditions of environmental hygiene, health care, and sanitation.

Considering the high likelihood of infection risk behavior engagements that could go on among prison inmates as well as the prevalent deplorable environmental conditions, it is conceivable that the prison setting could serve as a fertile ground for the breeding and spreading of infectious disease pathogens, of which HBV is key. This study is therefore important, in that the prisoners are only serving jail terms after which they would be released. As such, infected inmates who get released from jail could serve as reservoirs for spreading the virus and that could have dire consequences for the nation’s public health system. This study, among others, was aimed at bringing to the fore the HBV infection status in the prison facility, and underscoring the threat that the undiagnosed and uncontrolled spread of HBV infection could pose to the larger population of the country, given the risk of cancer and related health problems that are associated with HBV infection. As knowledge of HBV transmission in key populations is crucial, and yet there is no known study on HBV occurrences in Liberia prisons, this study was conducted to document the prevalence of HBV among prisoners at Monrovia Central Prison.

## 2. Materials and Methods

### 2.1. Study Site, Sample Size, and Study Population

A cross-sectional study was carried out at the Medical Laboratory of the JFK Medical Hospital and the Health Center of the Monrovia Central Prison, both in Monrovia, Liberia, and the National Public Health Reference Laboratory in Korle Bu, Ghana. The study participants were male and female prisoners between the ages of 19 and 55. The Monrovia Central Prison was chosen because it is the largest and the most crowded prison in Liberia and it houses more than 50% of the country’s prison’s total population. All inmates who qualified and were willing to participate were included, except those who were not willing to allow a blood draw. The number of those enrolled for the study was determined from the formula *N*= Z^2^pq/d^2^, as provided by Mugenda and Mugenda [12], where Z is the standard normal deviation at the required confidence interval of 95% (1.96), p being the estimated prevalence (6.12%) based on a previous study [13], with d as the margin of error given as 0.05, and q given as 1 − p. The computation gave *N* to be approximately 89, which was rounded up to 100.

### 2.2. Ethical Approval and Informed Consent

The study was approved by both the Institutional Review Board (IRB) of the University of Liberia and the Ministry of Justice, which is in charge of prisons. The study was explained to all participants and those who understood and were willing to participate voluntarily gave written informed consent before blood draw. Samples collected were deidentified to all others except the principal investigator for the purposes of giving feed-back to participants when necessary.

### 2.3. Sample and Data Collection

Participants’ sociodemographic data were obtained using a semi-structured questionnaire. Data collected included age, sex, marital status, educational background, and religion, among others. Subsequently, 4 mL of blood samples was carefully taken by venipuncture from each of the 100 prison participants into EDTA anticoagulant tubes. Blood samples were centrifuged and plasma was harvested and stored at −20 °C until use.

### 2.4. Serological Profile Assay

Plasma samples were screened for hepatitis B serological markers, namely HBsAg, HbsAb, HbeAg, HbeAb, and HbcAb, using a qualitative rapid HBV assay kit (MicroPoint Bioscience Ltd., Santa Clara, CA, USA) for hepatitis B profile. The assay was performed according to the manufacturer’s instructions.

Based on the outcome of the HBV serological profile assay, samples were further categorized into 5 based on evidence of the following: (a) active HBV infection and viral replication (positive HBsAg and positive HBeAg); (b) active HBV infection but non-replicating virus (positive HBsAg and negative HBeAg); (c) resolved HBV infection or natural immunity (negative HBsAg, negative HBeAg, negative HBeAb, positive HBsAb, and positive HBcAb); (d) HBV vaccinated (negative HBsAg, negative HBeAg, negative HBeAb, positive HBsAb, and negative HBcAb); and (e) susceptibility to HBV infection/HBV naïve (negative HBsAg, negative HBeAg, negative HBeAb, negative HBsAb, and negative HBcAb).

Further categorization was conducted and analyzed for statistical relevance with focus on (1) HBV infection (active infection: HBsAg positive; non-active infection: HBsAg negative) and (2) HBV exposure (exposed: actively infected and resolved HBV infection/natural immunity; HBV unexposed: HBV susceptible/naïve and HBV vaccinated).

### 2.5. Molecular Assay

Following the serological analysis of the HBV markers, a quantitative PCR (qPCR) assay was performed on 28 selected samples representing the 5 serological categories to confirm the presence or absence of HBV DNA and to estimate the amount of virus (viral load) in the test samples.

#### 2.5.1. DNA Extraction

DNA extraction was performed using the RADI PREP DNA/RNA kit (KH Medical Co., Ltd., Gyeonggi-do, Republic of Korea) and essentially followed the manufacturer’s instructions.

#### 2.5.2. DNA Amplification

HBV amplification and quantification were achieved by the use of the Bosphore multiplex real-time PCR kit (Anatolia Geneworks, Istanbul, Turkey), a commercial kit that detects and estimates hepatitis B viral DNA concentration in human serum or plasma. The PCR reagents and serum/plasma standards for virus quantification have been designed to meet WHO standards (NIBSC Code 971750), and are useful for the detection of HBV genotypes, A to H. The linear range of quantitation is from 1 × 10^1^ to 1 × 10^9^ IU/mL (1 logIU/mL to 9 logIU/mL) and the lower detection limit for the assay is 1 × 10^1^ IU/mL (1 logIU/mL) [14]. As the Bosphore kit’s assay is based on the real-time PCR approach, a linear relationship exists between amplification cycle threshold (Ct) values and starting amounts of target nucleic acid/standards [14]. Hence, the concentration of starting nucleic acid of the target is deducible from standard curves generated with Ct values of standards with known starting nucleic acid concentrations.

Each sample PCR reaction was set up to contain 5 μL plasma sample/standard/control, 15 μL PCR Master mix, 0.1 μL internal control, and made up with nuclease-free water to 25 μL reaction volume. Note that as and when necessary, the above mentioned volumes were varied uniformly to increase reaction sensitivity. PCR cycling conditions were in accordance with the manufacturer’s instructions. Ct values and standard curves were generated using the Magnetic Induction Cycler software from International Maritime Hospital, Ghana. Samples were considered ‘HBV target detected’ and viral load was subsequently quantified when amplification curves for the target were evident with Ct values less than or equal to 40.

### 2.6. Statistical Analysis 

Detailed statistical analysis was performed using DATA*tab* online statistics calculator (Datatab.net), GraphPad Prism 9.4.1 (https://www.graphpad.com, accessed on 20 October 2022), and Stata/MP 14 (https://www.stata.com/stata14/, accessed on 31 October 2022). To assess potential associations between categorical variables, Fishers’ exact test or Chi-squared analysis were used where appropriate. Logistic regression models were performed to determine potential predictors of HBV active infection and HBV exposure. An independent *t*-test was used to assess the difference in mean values between two independent variables. For all analyses, a *p*-value of less than 0.0500 was accepted as significant.

## 3. Results

### 3.1. Participants’ Demographic Characteristics, Knowledge, and Practices

One hundred prisoners were studied at the Monrovia Central Prison. The study involved screening for the hepatitis B virus infection, assessing the nature or extent of infectivity in positive individuals, and evaluating the viral load in actively infected individuals. One hundred inmates comprising 76 (76.0%) males and 24 (24.0%) females took part in the study. Most participants (44, 44.0%) were below 31 years while less than a third (26.0%, *n* = 26) were above 40 years. An appreciable number (76, 76.0%) of the respondents had a minimum of Junior High School (JHS) level of education. The findings obtained showed that the majority (76.0%, *n* = 76) of the respondents were married and more than half (66.0%) were unemployed prior to incarceration. Details of the demographic characteristics of participants are shown in Table 1A.

With regard to knowledge about hepatitis B infection, social life and practices that might constitute a risk of infection (Table 1B), 16.0% (*n* = 16) out of the number studied had adequate knowledge about HBV infection and 9 indicated that they had been vaccinated against HBV infection. The majority (74.0%, *n* = 74) of the respondents had multiple sexual partners; thirty-three (33.0%) used intravenous drugs and at least half (56.0%) of the respondents had tattoos, while seven had previously received a blood transfusion.

### 3.2. Demographic Characteristics and Active HBV Infection

HBsAg seroreactivity was considered to indicate active infection. By this criterion, the study found 14 (14%) participants with active HBV infection. Analysis was also performed to find possible associations between demographic characteristics and active HBV infection. There were more females (16.7%) with active HBV infection compared to males (13.2%). This difference was, however, not statistically significant (*p* = 0.738). However, the level of education appeared to be a crucial factor with regard to active HBV infection. A statistically significant difference was observed between those who had only an elementary level of education or below (29.2%), and those who were educated above the elementary level (9.2%, *p* = 0.037).

Furthermore, a binary regression to determine the factors influencing active HBV infection showed that only a higher odds of no education/elementary education (OR = 7.51) was statistically significant (*p* = 0.009). It is noteworthy that none of those who reported having been vaccinated had active HBV infection, while 15.4% of non-vaccinated respondents were actively infected.

### 3.3. Categorization and Characterization of Hepatitis B Serological Markers

In general, exposure to HBV was decided by HBsAg seropositivity (active infection) as well as evidence of resolved infection/natural immunity. By this criterion, the findings showed that about a third (34.0%) of the study participants had been exposed to HBV.

The number of individuals belonging to the five serological categorizations have been shown in Table 2. The prevalence of active infection with replicating virus in women was 4.2%. Active infection with non-replicating virus observed was 12.5% and the prevalence of resolved infection was 16.7%. The total exposure prevalence among women and men were 33.4% and 34.3%, respectively. Serological results showed that 7.9% of the men in the study were vaccinated, and 4.2% of the women were vaccinated. It is worthy of note that, the prevalence of HBV exposure among inmates with a history of IV drug use was 45.5%, while among those with no history of IV drug use was 28.4%. Additionally, worthy of note is the overall exposure prevalence for those with a history of blood transfusion was much higher than for those with no history (71.4% versus 31.2%).

Representative samples from all five serological categories were drawn and tested by qPCR for possible detection of HBV DNA. This yielded positive results from the first two categories only. HBV DNA was detected in all five samples from the actively infected with replicating virus category. However, HBV DNA was detected in only two out of the nine samples from the actively infected with a non-replicating virus category. The viral loads for samples with detected HBV DNA ranged from 1.43 to 4.45 Log IU/mL, with an average of 2.92 Log IU/mL for the category of active infection with replicating virus and 1.795 Log IU/mL for the non-replicating virus infection category. The difference in viral load between the two categories was, however, not statistically significant. Two of the samples with detectable DNA had simultaneous detection of HBsAg and HBsAb.

### 3.4. Statistical Associations between Demographic Characteristics and Risk Factors for Exposure to Hepatitis B Virus Infection

Among the various indicators tested, the statistical analysis underscored a significant association between a history of blood transfusion, vaccination status, and HBV exposure, where exposure refers to either an active or resolved infection. By logistic regression analysis, the outcome of both bivariate (5.517, *p* = 0.049) and multivariable analyses (6.89, *p* = 0.034) indicated that the odds of exposure to HBV infection increased significantly with a history of blood transfusion. Significant odds (2.595, *p* = 0.049) were also obtained through a multivariable analysis, which showed that a history of IV drug use increased HBV exposure among the prison inmates studied.

## 4. Discussion

A prison or jail is a facility that houses people of different backgrounds and social status/orientation for various reasons: punitive, correctional, or pending determination of a case. This includes those who are in the process of getting arrested, being tried, being sentenced, or who are serving jail terms [15]. Conditions that prevail, and activities that go on in the prison environment may make the prison serve as a breeding ground for diseases transmission from and among inmates carrying infectious pathogens [16]. This study sought to determine the prevalence or potential for transmission of hepatitis B infection among the prison inmates at the Monrovia Central Prison in Liberia.

The study recorded a prevalence of 14% active HBV infection among the inmates studied. The prevalence recorded indicates that HBV was highly endemic among the population studied, and to some extent, this might reflect the situation in Liberia. This prevalence among the Monrovia prison inmates was higher than that (6.1%) obtained among health workers at J.F.K Hospital [13] and that of the 3.3% prevalence reported by Fardolo et al. [17] among the voluntary blood donors at the Telewoyan Hospital in Voinjama, Lofa county, Liberia.

Invariably, prison facilities are expected to have a higher prevalence of blood-borne diseases such as HBV due to the unsafe practices of the incarcerated inmates. Even though the prevalence was high and comparable to the 13.1% prevalence seen in male prison inmates in Taiwan, for example [18], the prevalence observed in this study was lower than that reported in two neighboring West African countries, Ghana and Nigeria, where rates of 17.4% and 25.5% in Ghana [19,20], and 18% and 23% in Nigeria [21,22] have been reported. It is noteworthy that the sample size of this study was smaller as compared to those of the Ghana and Nigeria studies cited [19,20,21]. In general, however, the HBV prevalence in Liberia may be truly lower, but it was higher when compared with the prevalence from similar studies conducted in Rwanda or India, which reported a prevalence of 4.3% [23] and 8% [24], respectively. Furthermore, an extremely low prevalence of 0.9% has been reported among jail inmates in the USA [15], which could be attributed to the institutionalized measures of infection prevention and control programs besides the other interventions in place in American correctional facilities [25]. Hence, despite possible differences in sociocultural background and practices, it is conceivable that the effectiveness of infection prevention and control measures could play an important role in the HBV transmission among prison inmates.

The active infection prevalence rate was observed to be a little higher in females (16.7%) compared to males (13.2%). Even though the difference was not statistically significant, it nevertheless agrees with the findings by Taura et al. [26] who found that females had the highest prevalence of HBV infection. On the other hand, Lawal et al. [27] reported a higher prevalence of HBV infection in males than in females. Again, this difference must be considered along with possible sociocultural backgrounds and practices in mind, besides the sample size. Women’s care roles and vulnerability to sexual violence [28,29] could potentially put them at a higher risk of infection, especially in the prison environment, where their vulnerability is at a higher risk. Further studies that critically interrogate how sociocultural factors, such as gender, drive HBV are needed.

To examine the potential transmissibility of the virus in various groups of seropositive individuals, a molecular technique was employed since such techniques are known to be very sensitive [30]. HBV DNA was detected in all five participants who were found to be actively infected (HBsAg seroreactives), with serological evidence of actively replicating virus, by virtue of HbeAg seropositivity. In contrast, HBV DNA detection was successful for only two out of nine of those actively infected (HbsAg seroreactives), but with negative HbeAg (non-replicating virus) results. This finding about the utility of HbsAg and HbeAg serological markers in identifying potential transmitters of the virus is in synchrony with previous reports [31]. Additionally, a viral load of greater than 3.30 logIU/mL (that is, 2000 IU/mL) is known to be a strong risk predictor of hepatocellular carcinoma, independent of HBeAg status, liver cirrhosis, or alanine aminotransferase levels [32]. The findings in this study revealed that two participants had viral loads greater than 3.30 logIU/mL. This is a concern that warrants some interventions and underscores the need for screening in order not to aggravate the plight of incarcerated individuals. Additionally, two of the participants with detectable DNA interestingly had simultaneous detection of HBsAg and HBsAb. This is a phenomenon that has been observed by others in the literature, for about 10 to 25% of chronic carriers with sequencing results of such carriers suggesting selection of HBV immune escape mutants during the chronic infection stage [33,34].

What is also critical is the fact that 34% of the participants had been exposed to the hepatitis B virus (20% recovered with natural immunity and 14% were actively infected). This may suggest that HBV exposure in Monrovia Central Prison is high. It was, however, not determined whether the exposure to HBV infection occurred while incarcerated or before incarceration. The study nonetheless demonstrates the high possibility of the spread of the virus among prison inmates if risk awareness and safe social practices as well as control and intervention measures such as vaccination, are not implemented. This is also of concern, noting that the serological evidence of vaccination was found for as low as 7% of the participants. Blood transfusion was found to be a significant predictor of HBV exposure. This is in agreement with the general knowledge that hepatitis B is a transmission-associated infection [21,35,36]. This risk has been attributed to the occult carriage of HBV infection, infection with immunovariant viruses, and the pre-seroconversion window period of blood donors [35]. As such, in medical interventions that require blood transfusions, which may constitute a major risk for transmission of blood-borne infections, the use of very sensitive methods for screening blood and blood products is crucial. The history of IV drug use was a significant predictor of HBV exposure, which is consistent with previous reports [37,38,39,40,41]. These findings underscore the need to enforce measures to control such risky practices. Focusing on improving the level of education appears to have promises of improving knowledge and potentially reducing transmission of infectious pathogens, including HBV, in Monrovia, based on the finding that HBV was mostly prevalent among the least educated. Further studies using a larger sample size and involving various key populations need to be performed to clarify these findings.

This study had some limitations. First, the prisoners studied were not screened for the hepatitis B virus before incarceration. This, however, falls outside the scope of this study. It was therefore not possible to determine what percentage of the infected persons acquired the infection in prison. Secondly, data were not collected on the length of stay in prison, which could give possible clues on whether inmates were infected before incarceration. Lastly, the sample size for the study was small, and therefore, it lacked sufficient statistical power to allow definite conclusions to be made. Future studies should address these limitations for better outcomes.

## 5. Conclusions

This study documents the presence of infectious HBV among prison inmates in the Monrovia Central Prison. The prevalence observed indicates that HBV is highly endemic among the inmates at the Monrovia prison. The study further noted risky practices among the inmates that could fuel the transmission of the virus; however, only a few had been vaccinated against HBV. This study is important not only for the good of the study population and the people of Liberia in general but also has transmissibility implications for neighboring countries due to the frequent and usually unrestricted mobility between and among West African countries. These findings should call for policies that make HBV vaccine administration to prison inmates imperative, ensuring that such vaccination programs are complete since incomplete vaccination could also lead to a rapid decline in HBV immunity [42]. To the best of our knowledge, this study represents the first of its kind to document the prevalence of HBV infection in the Liberian prison population. The accumulation of these data would be useful for the hepatitis B virus control and public health safety assurance efforts in Liberia.

## Figures and Tables

**Table 1:** Participants’ demographic characteristics, knowledge, and practices.

A	Demographic Characteristics (*N* = 100)										
Gender		Age Group (years)			Educational Level		Marital Status		Occupation		
Male	Female	<30	31–40	>40	None/Elementary	JHS/SHS/Tertiary	Married	Single	Employed/Farming	Unemployed	Other
76	24	44	30	26	24	76	24	76	19	66	15
									*Other includes students*		
**B**	**Knowledge and Practices**										
**HBV Knowledge**		**HBV Vaccinated**			**Intravenous Drug Use**		**Blood Transfusion**		**Number of Sexual Partners**		
Yes	No	Yes	No		Yes	No	Yes	No	Multiple	One	N/A
16	84	9	91		33	67	7	93	74	24	2
									*N/A = no response*		

**Table 2:** Seroprevalence of the different HBV infection categories.

Characteristics	Total	Active & Replicating	Active & Non-Replicating	Resolved/Natural Immunity	Vaccinated	Susceptible/ Naïve
	*N*	*n*(%)	*n*(%)	*n*(%)	*n*(%)	*n*(%)
	100	5(5.0)	9(9.0)	20(20.0)	7(7.0)	59(59.0)
**Sex**						
Male	76	4(5.3)	6(7.9)	16(21.1)	6(7.9)	44(57.9)
Female	24	1(4.2)	3(12.5)	4(16.7)	1(4.2)	15(62.5)
**History of IV Drug Use**						
Yes	33	2(6.1)	3(9.1)	10(30.3)	1(3.1)	17(51.5)
No	67	3(4.5)	6(9.0)	10(14.9)	6(9.0)	42(62.7)
**HB Vaccination Status**						
Yes	9	0(0.0)	0(0.0)	0(0.0)	7(77.8)	2(22.22)
No	91	5(5.5)	9(9.9)	20(22.0)	0(0.0)	57(62.6)
**Blood Transfusion History**						
Yes	7	0(0.0)	1(14.3)	4(57.1)	1(14.3)	1(14.3)
No	93	5(5.4)	8(8.6)	16(17.2)	6(6.5)	58(62.4)

## Data Availability

Serological data generated and presented in this study are available on request from the corresponding authors.
